# Action Spectroscopy of Mass‐Selected Ions—From Principles to Applications

**DOI:** 10.1002/jms.70118

**Published:** 2026-09-30

**Authors:** Pedro Henrique Martins Garcia, Gustavo Cervi, Cesar Augusto Gonçalves Dantas, Guilherme Obeid, Thiago C. Correra

**Affiliations:** ^1^ Department of Fundamental Chemistry, Institute of Chemistry University of São Paulo São Paulo São Paulo Brazil

## Abstract

Gas‐phase ion spectroscopy provides direct, structure‐sensitive information that complements and extends conventional mass spectrometry workflows, offering structural resolution that accurate mass and fragmentation patterns alone cannot always achieve by themselves. This review offers a practical entry point into the field by showcasing its analytical capabilities, addressed to mass spectrometrists and researchers from adjacent disciplines for whom ion spectroscopy represents an underexplored analytical resource. Four representative techniques are discussed: Electronic UV/Vis photodissociation (UVPD), infrared multiple photon dissociation spectroscopy (IRMPD), vibrational tagging photodissociation ion spectroscopy (IRPD), and helium nanodroplet isolation infrared spectroscopy (HENDI‐IR). For each, the underlying physical principles are briefly introduced, and representative applications are presented with emphasis on problems of direct analytical relevance, including the structural characterization of isomeric metabolites, reactive intermediates, glycoconjugates, and drug‐related compounds. Applications integrating ion spectroscopy with liquid chromatography (LC) and ion mobility spectrometry (IMS) are also discussed. The goal of this review is to convey, through carefully chosen examples, the breadth of problems that gas‐phase ion spectroscopy is uniquely positioned to solve and to lower the threshold for its adoption by a wider scientific community.

## 1 Introduction

Mass spectrometry has become one of the most powerful analytical tools in modern chemistry and biochemistry, capable of detecting and identifying thousands of compounds from complex mixtures in a single experiment in the most challenging matrices and conditions. Yet, a sobering result published in 2024 by Van Tetering et al. exposes a fundamental limitation of the approach [1]. When fragment ion annotations from three major MS/MS spectral libraries were tested against spectroscopically established identifications, the vast majority were found to be incorrect, with errors arising primarily from rearrangements that conventional *tandem* MS cannot differentiate. This finding is not a critique of mass spectrometry, but a clear illustration of the limits imposed by accurate mass and fragmentation pattern alone, and a compelling argument for the need for complementary structural tools.

This role can be fulfilled by the application of spectroscopic methods to mass‐selected ions in the gas phase, as it provides direct structure‐sensitive information that extends well beyond what exact *m*/*z* and dissociation patterns can reveal.

The relevance of ion action spectroscopy for structural information has been established across a broad range of chemically relevant problems. In the structural characterization of carbohydrates and glycoconjugates, where isomeric complexity renders MS/MS‐based identification unreliable, gas‐phase infrared and UV spectroscopy have enabled unambiguous assignment of linkage position and stereochemistry [2]. In catalysis and the study of reaction mechanisms, ion action spectroscopy has allowed the direct structural characterization of reactive intermediates and transient species that cannot be isolated by other means [3]. In metabolomics, infrared ion spectroscopy has provided orthogonal structural resolution for indistinguishable isomers, including the identification of disease biomarkers [4, 5, 6]. These examples reflect a consistent capacity to address structural questions that fall outside the reach of conventional MS workflows.

The increasing accessibility of ion spectroscopy instrumentation, including benchtop laser sources and commercial platforms adapted for photodissociation experiments, has substantially lowered the barriers to adoption that historically confined the technique to specialized physical chemistry laboratories. Nevertheless, the application of these techniques is still not mainstream and relies on a relatively small portion of the mass spectrometry community [7].

In this context, this review is addressed to mass spectrometrists and researchers from adjacent disciplines like pharmacology, clinical biochemistry, catalysis, for whom ion spectroscopy and photodissociation represent a largely unexplored analytical resource. Rather than surveying the historical development of the field or exploring all available methods, both of which have been treated thoroughly elsewhere [4, 8, 9, 10, 11, 12, 13, 14], the present work offers a practical, application‐oriented introduction organized around four representative techniques: electronic UV/Vis photodissociation (UVPD), infrared multiple photon dissociation spectroscopy (IRMPD), vibrational tagging photodissociation ion spectroscopy (IRPD), and helium nanodroplet isolation infrared spectroscopy (HENDI‐IR).

These specific approaches were selected because they collectively illustrate the fundamental principles governing gas‐phase photodissociation of mass‐selected ions, and because their application examples span a broad and representative range of analytical problems and represent the advances currently being witnessed in the field. More specialized methods and their combinations are introduced where directly relevant to a specific application. The analysis of complex matrices and hyphenated workflows combining ion spectroscopy with liquid chromatography (LC) or ion mobility spectrometry (IMS) is discussed in a dedicated section. The relevant role of theoretical methods and simulations to support spectral assignment will not be covered in depth in this review, as it has been discussed elsewhere [15, 16, 17, 18]. An overview of commercially available instruments and dedicated facilities rounds out the discussion, followed by concluding remarks on the outlook for broader adoption of these methods.

## 2 Principles of Ion Action Spectroscopy

To perform spectroscopy on gas phase ions an ion population must interact with electromagnetic radiation, and such radiation should be capable of promoting a measurable change in the system. In principle, the absorbed radiation could be quantified by the difference between incident and transmitted intensities, as in conventional absorption spectroscopy. In practice, however, this approach is not feasible. The number of ions in mass spectrometers typically fall in the 10^8^ range, orders of magnitude below even the most dilute solutions routinely analyzed by absorption techniques [19]. The optical path lengths and ion densities achievable in gas‐phase experiments are simply insufficient to produce a measurable attenuation of the radiation beam. The spectroscopic information must therefore be extracted indirectly, by monitoring the *effect, or action*, of the radiation on the ion population rather than its *absorption*. It is this reliance on a measurable consequence of photon absorption that defines the approach and names it *action spectroscopy* [11].

In the case of the action spectroscopy of mass‐selected ions the most common effect used to quantify the absorption of radiation is some sort of dissociation, regardless of the spectral range employed, despite some inherent differences in the mechanism that lead to dissociation.

Therefore, by tuning the photon energy, the absorption of a resonant photon can take place, and the amount of dissociation its absorption causes at each wavelength can be correlated to the presence of an absorption band, generating an action spectrum of the ion. Despite some different approaches [20], the amount of photofragmentation can be calculated by comparing the intensity of precursor and fragment ions and is used to generate an action spectrum.

A shift from the infrared to the visible (and any other) spectral domain implies a change between distinct spectroscopic regimes governed by different selection rules and interaction mechanisms. Therefore, different photophysical mechanisms take place depending on the spectral range being evaluated and the specific action ion spectroscopy implemented.

The four implementations discussed in this review are represented in Figure 1 and will be discussed in the next sections.

**FIGURE 1:**
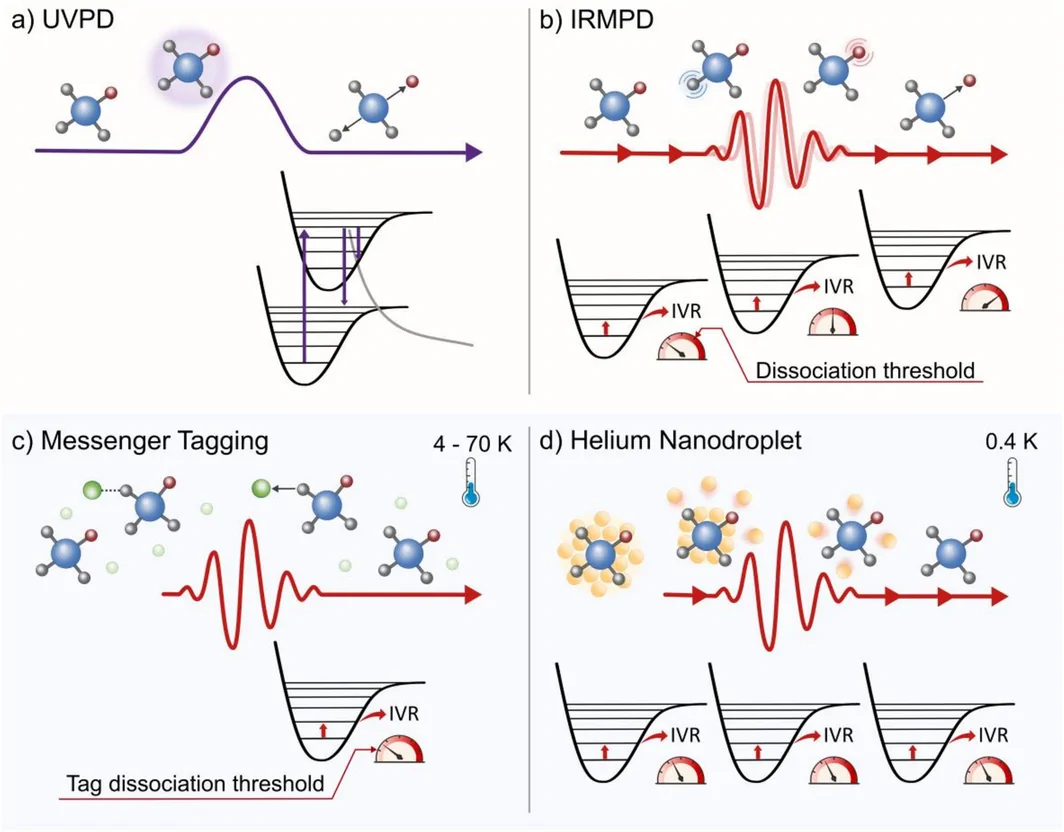
Representation of the four distinct techniques discussed. (a) Electronic UV/Vis photodissociation (UVPD), (b) infrared multiple photon dissociation (IRMPD), (c) vibrational tagging photodissociation represented for He, and (d) helium nanodroplet isolation spectroscopy. Arrowheads indicate the number of photons absorbed in each process (one in a and c, several in b and d). The shape of each radiation profile reflects the underlying level structure being probed. The Gaussian envelope in a represents the broadened, unresolved manifold of electronic levels observed at room temperature, while the wavy profiles in b, c, and d reflect the finer, discrete vibrational level structure and the vibrational broadening experienced in IRMPD.

### 2.1 UV/Vis Photodissociation

UV photodissociation spectroscopy exploits electronic transitions, in which absorption of a UV photon promotes the ion from its ground electronic state to an excited state (Figure 1a). Provided the photon energy exceeds or, following internal conversion, allows access to the dissociation threshold of the ion, fragmentation occurs allowing its detection as a function of laser wavelength [21, 22]. Because UV photons carry sufficient energy to drive dissociation in a single absorption event, the photon flux requirements of UVPD are comparatively modest, and dissociation can be achieved without the high fluence sources demanded by multiple‐photon schemes. This does not mean that the experiment itself is undemanding as laser alignment, synchronization with the ion manipulation sequence, and the instrument modifications required to introduce the beam into the trapping region remain nontrivial, as for the other implementations discussed here. The resulting spectra report on the electronic structure of the ion and, at room temperature, typically appear as broad, unresolved envelopes. For this reason, many room temperature UVPD studies explore the different fragments generated by UVPD when compared with other dissociation methods such as collision‐induced dissociation (CID) and other slow heating methods [23, 24].

The spectroscopy at the UV range when conducted at reduced/cryogenic temperatures substantially narrows the transitions and reveals detailed structural information on the ion [25, 26].

Optical parametric oscillators (OPOs), sometimes coupled with optical parametric amplifiers (OPAs), provide broadly tunable, high‐intensity radiation spanning the UV to the far‐IR. These systems exploit nonlinear optical processes in suitable crystals. Due to their broad tunability and benchtop footprint, OPO/OPA systems have become among the most widely used radiation sources in action ion spectroscopy both in the UV and IR range.

### 2.2 Infrared Multiple Photon Dissociation

Unlike in the UV range, photon absorption in the infrared range promotes vibrational transitions that, individually, do not deposit enough energy to promote ion dissociation, as the photon energy is far below the dissociation threshold of most covalent bonds [12]. Dissociation by this spectral range is usually achieved through sequential absorption of multiple photons (Figure 1b). After each absorption event, an intramolecular vibrational redistribution (IVR) event takes place, which relaxes the initially excited mode into the bath of the remaining vibrational modes and restores the resonance condition of the absorbing oscillator for further absorption. The cumulative internal energy eventually exceeds the dissociation threshold, producing fragment ions whose yield as a function of IR wavelength reflects the vibrational absorption profile of the precursor. Because this multiple photon process requires high photon flux to drive efficient dissociation, IRMPD has historically depended on high‐power infrared sources like free‐electron lasers (FELs), which impose significant infrastructure constraints on its implementation, and OPO/OPA systems that have limited flux and are typically dedicated to the high wavenumber range (> 2200 cm^−1^), even considering continuous or high repetition rate modern systems based on periodically poled crystals [27, 28].

The multiple‐photon nature of the IRMPD process carries consequences that must be taken into account when spectra are interpreted. The measured fragmentation yield and IRMPD band intensities do not compare directly with absorption intensities obtained from calculated spectra. Weak transitions may be suppressed and intense bands can appear broadened at high laser fluence. Larger ions with many vibrational degrees of freedom and high dissociation threshold systems may experience poor fragmentation. In practice, the comparison between an IRMPD spectrum and a calculated one is most reliable in terms of band positions and overall pattern rather than in terms of quantitative relative intensities [6, 29].

Usually, FELs are used for the lower wavenumber range owing to their exceptional photon flux and broad spectral tunability in the infrared fingerprint region. Unlike conventional laser sources, FELs generate coherent radiation by accelerating relativistic electrons through periodic magnetic structures, where the resulting oscillatory motion produces intense, tunable emission. Access, however, is constrained by the accelerator infrastructure required for their operation, limiting experiments to dedicated user programs at a small number of installations worldwide [30, 31, 32, 33, 34, 35].

### 2.3 Messenger Tagging Spectroscopy

The dependence of IRMPD on high‐power sources arises fundamentally from the need to accumulate sufficient internal energy to break a covalent bond, an event that competes with the deactivation by collision against the backpressure of the instrument [36].

Messenger tagging spectroscopy circumvents this limitation by replacing the covalent dissociation event with the elimination of a weakly bound neutral tag, typically a noble gas atom (He, Ne, Ar) or a small molecule (H_2_, N_2_, H_2_O), noncovalently attached to the ion of interest (Figure 1c) [37, 38]. As the binding energy of these complexes is smaller than that of a single IR photon in the relevant spectral range, absorption of a single photon is sufficient to dissociate the complex and release the tag. This single‐photon sensitivity dramatically lowers the required laser power and, critically, makes tagging spectroscopy compatible with compact, tunable benchtop sources such as OPOs, even at lower wavenumbers (< 2000 cm^−1^). Nevertheless, to allow a neutral tag to be weakly bound to the ions, it is usually necessary to reach cryogenic temperatures, which, in turn, minimize spectral congestion and yield sharper, better‐resolved spectra than room‐temperature IRMPD. It should be pointed out that the ion–tag interaction may affect the structure of the ion and consequently the recorded spectra. Helium is usually the least perturbing option, at the cost of demanding the lowest temperatures and low adduct signals. Heavier noble gases and molecular tags bind more strongly and are correspondingly easier to form, but they may shift vibrational frequencies, broaden transitions, and, where the tag competes with an existing intramolecular interaction, alter the relative populations of conformers or even the structure itself. The effect is most severe for ions in which the tag interacts directly with the site under interrogation, and least severe for large, rigid ions where the tag binds peripherally. This is a particular concern with water tags, which have higher binding energies and therefore do not require the very low temperatures needed for noble‐gas adduct formation. Interestingly, this approach has been used implementing water and other solvents as tags to allow the correlation of the gas‐phase studies with solution phase behavior [38, 39]. Comparative measurements using different tags on the same system remain the most reliable way to establish whether an observed feature is intrinsic to the ion or induced by the tag [40].

### 2.4 Helium Nanodroplet Spectroscopy

Helium nanodroplet isolation spectroscopy represents the most extreme implementation of the cryogenic principle. Rather than forming discrete ion–tag adducts, the analyte ion is embedded within a large droplet of superfluid helium containing typically 10^3^–10^6^ atoms, which maintains the ion at an equilibrium temperature of approximately 0.4 K (Figure 1d). At this temperature, the ion is effectively confined to its vibrational ground state and the thermal population of excited vibrational levels becomes negligible, which largely suppresses thermal broadening and greatly reduces conformational heterogeneity [41].

Despite some dispute on the actual mechanism [42, 43, 44, 45], the spectra acquisition process can be described as initiated by the absorption of the photon by the embedded ion that transfers energy to the surrounding helium atoms, leading to the eventual release of the ion. This event indicates absorption of radiation and can be easily followed by the mass spectrometer. The resulting spectra achieve a level of resolution and conformational selectivity that is unattainable by any of the preceding approaches, at the cost of greater instrumental complexity.

### 2.5 Comparison of the Action Ion Spectroscopy Schemes

The four implementations described above differ substantially in the structural information they deliver and in the experimental effort they demand, and the choice between them is in practice dictated by the kind of structural information necessary. Table 1 summarizes the main aspects and limitations of each approach.

**TABLE 1:** Comparative summary of the four action ion spectroscopy implementations discussed. Values are indicative and intended to guide the choice of technique rather than to define strict operational limits, because performance depends strongly on the specific ion, instrument, and radiation source employed.

Technique	Transition probed and typical range	Instrumental requirements	Throughput and advantage	Principal limitations
UV/Vis photodissociation (UVPD)	Electronic; ca. 200–800 nm	Tabletop pulsed lasers (Nd:YAG‐pumped OPO, excimer)	Moderate; single‐photon process, useful fragmentation scheme	Requires a chromophore; room‐temperature spectra are congested.
Infrared multiple photon dissociation (IRMPD)	Vibrational; ca. 600*–4000 cm^−1^	OPO/OPA or CO_2_ lasers above ca. 2200 cm^−1^. *FEL for the fingerprint region;	Moderate; room‐temperature operation	Propense to shifts, transparency and power broadening; fingerprint access is FEL‐dependent
Messenger tagging (IRPD)	Vibrational; ca. 600–4000 cm^−1^	OPO/OPA (FEL can be used but is not necessary); requires a cryogenic approach and available tags	Low; allow fingerprint with tabletop radiation sources	Tag may shift frequencies and alter conformer populations and reduces ion yield
Helium nanodroplet isolation (HENDI‐IR)	Vibrational; ca. 600–4000 cm^−1^	Dedicated droplet source and pickup region coupled to the mass spectrometer	Lowest; highest spectral resolution	Low duty cycle, few instruments available

Broadly, UVPD offers the most accessible instrumentation but requires a chromophore and yields congested spectra unless cryogenic cooling is implemented; IRMPD provides general applicability at the cost of spectral broadening and facility‐dependent access for the fingerprint region; messenger tagging recovers narrow bands at the cost of cryogenic operation and is prone to tag effects; and helium nanodroplet isolation delivers the highest attainable resolution while demanding the most specialized apparatus. It is also worth noting that these techniques are complementary rather than mutually exclusive, and that several of the studies discussed in the following sections may combine more than one of them, to address a single structural problem.

### 2.6 The Role of Theoretical Calculations

A further point deserves explicit mention before turning to applications. With the exception of the cases in which a reference standard is available, the structural assignment of an action spectrum usually depends on the comparison with calculated spectra for candidate structures. This comparison is only as reliable as the underlying computational protocol, which must include an adequate conformational search, an appropriate choice of functional and basis set, and, sometimes, treatments that go beyond the harmonic approximation. Relatively recent developments like automated conformational sampling, machine learning potentials, and the progressive construction of experimental spectral databases and automated spectral matching routines all reduce the effort required to assign specific structures and are likely to be decisive in determining how widely these techniques are adopted outside specialist laboratories in the near future. A detailed treatment of these computational aspects is beyond the scope of the present contribution, and the reader is referred to the dedicated literature [15, 16, 18, 46].

## 3 Applications

The following sections shift focus from principles to applications, examining how the discussed techniques have been applied in different selected fields. For each field, selected examples were chosen to illustrate the capabilities of each technique. A dedicated subsection then examines workflows in which ion spectroscopy is integrated with chromatographic techniques, reflecting the growing role of these methods within broader analytical pipelines. These fields were not chosen to be exhaustive, and many other areas of applications were not discussed in detail in the text, like food chemistry [47], bioactive molecules [41, 48, 49], agrochemicals and environmental chemistry [50], coordination chemistry [51, 52], and atmospheric and astrochemical applications [53, 54, 55].

### 3.1 Biomolecular and Disease‐Related Molecules

The use of UVPD at room temperature takes advantage of the fact that UV photons can induce different fragmentation pathways from other activation schemes such as CID and other slow heating implementations (Figure 2a). These unique fragmentations are especially relevant in the case of biomolecules such as nucleic acids, lipids, carbohydrates and proteins, because it allows a more precise characterization of these analytes [13].

**FIGURE 2:**
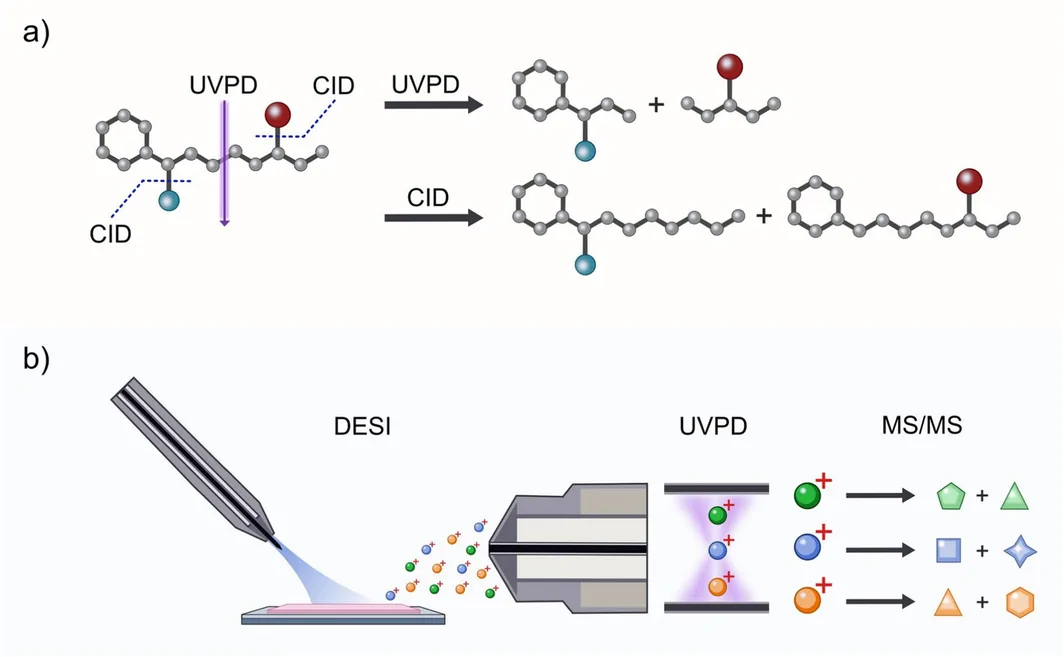
(a) Schematic representation of the different fragmentations generated by UVPD spectroscopy and other slow‐heating methods such as CID and IRMPD. More specifically, the representation shows that posttranslation modifications (PTM represented by the different spheres) can be located by UVPD that promotes the protein backbone breakage, while CID tends to cleave the modification itself. (b) Representation of the use of UVPD for differentiating diagnostic ions for the identification of diseases. In this case, the system is represented as interfaced with a desorption electrospray ionization (DESI) source representing the imaging abilities that can be coupled to ion photodissociation/spectroscopy approaches.

The ion fragmentation through UVPD has been reported as a method for the interrogation of protein structure and post‐translation modifications (PTM) combined with soft ionization sources that maintain the noncovalent characteristics in a protein.

The work of Brodbelt and coworkers [13] explores the use of UVPD as a fragmentation scheme for top‐down proteomic analysis on five model proteins. The use of UVPD for structural characterization has been shown to result in greater information for structure annotation and has inspired the development of a library for UVPD‐based MS/MS data [56].

Another interesting application of UVPD related to PTMs comes from the work of Fouque et al. [57], where a new workflow is presented based on the first step of fragmentation by UVPD of both intact and modified histones. The first takeaway presented by the authors is how UVPD can be attached to *tandem* mass spectrometry, in this case UVPD‐TIMS‐q‐ECD‐ToF MS/MS. The other is that the UVPD event can be useful prior or after other strategies, such as ion mobility on the toolbox of molecular analysis.

One representative example of the use of UVPD fragmentation has been developed, alongside desorption electrospray ionization source, for MS imaging of fatty acids from human tissue, essential for the formation of cellular membranes (Figure 2b) [58]. Moreover, the paper reports that cancerous cells display characteristic double bonds that were pinpointed through the unique characteristic of UVPD fragmentation, by cleaving the C–C bond adjacent to the double bond. The method was applied with DESI in an imaging workflow of breast tissue samples to identify fatty acid isomer abundance and outline tumor regions correlated to breast cancer [58].

Amino acids and glycans are frequently used to showcase the analytical capabilities of IRMPD [59, 60, 61, 62]. Beyond these model systems, the technique has also found application in clinical analysis [4] and in the study of disease‐related molecules.

Comprehending the structural diversity of peptides and high order oligomers by ion action spectroscopy is of particular interest for understanding the molecular processes involved in the onset of amyloid diseases such as Alzheimer's and Parkinson's diseases [63], as well as type II diabetes, because small soluble oligomers of amyloidogenic peptides have been linked to toxicity and onset of these diseases. Furthermore, specific glycan structures may further shape the protein physical properties through glycosylation [64].

The aggregation of VEALYL oligomers was evaluated by Bakels and coworkers [65] by the combined use of ion mobility and IRMPD spectroscopy. Their results show that the combined use of quadrupole mass filter and ion mobility allows the observation of oligomer‐specific IR features in the NH stretching vibrational region and to identify specific sialylated N‐glycan fragments from glycoproteins using their OH signatures.

Seo et al. [66], using IRMPD spectroscopy coupled to drift‐tube ion mobility spectrometry (DT‐IMS), evaluated the β‐sheet formation in oligomers of VEALYL and YVEALL peptides, chosen in this study due to their reported spontaneous formation of insoluble amyloid fibrils. The authors recorded IRMPD spectra at the fingerprint region using the FHI‐FEL free electron laser facility. The obtained data suggest that for these oligomers in scope, which consist of less than 10 peptides each, there is a proportionally high number of β sheets. IRMPD was also fundamental in allowing the authors to establish a relationship between an increase in the number of β sheets and an increase in the deviation between the measured collision cross‐section (CCS).

The use of helium nanodroplet spectroscopy for the evaluation of glycosaminoglycans [67], glycerophospholipid [68], and glycolipids [69] has also been reported. Kirschbaum and coworkers [69], for instance, characterized isobaric glycolipid overcoming analytical difficulties of standard MS and NMR, revealing different lipids, glycan headgroups, and configurations.

### 3.2 Pharmaceuticals and Clinical Applications

Besides the previous applications, other ion mobility schemes were also employed in conjunction with UVPD for the evaluation of pharmaceutical drugs.

Mashmoushi and coworkers [70] traced a quantitation problem to its origin, using differential ion mobility (DMS or FAIMS) coupled to room‐temperature UV spectroscopy (Figure 3a) to resolve the isomeric ion populations of protonated Rivaroxaban, an anticoagulant drug. These techniques allowed the differentiation of populations related to the bare and cluster adducts of the precursor ions, suggesting that during MRM detection of this drug, fluctuations in the ion nature could lead to diminished or enhanced intensities, generating incorrect quantitation results.

**FIGURE 3:**
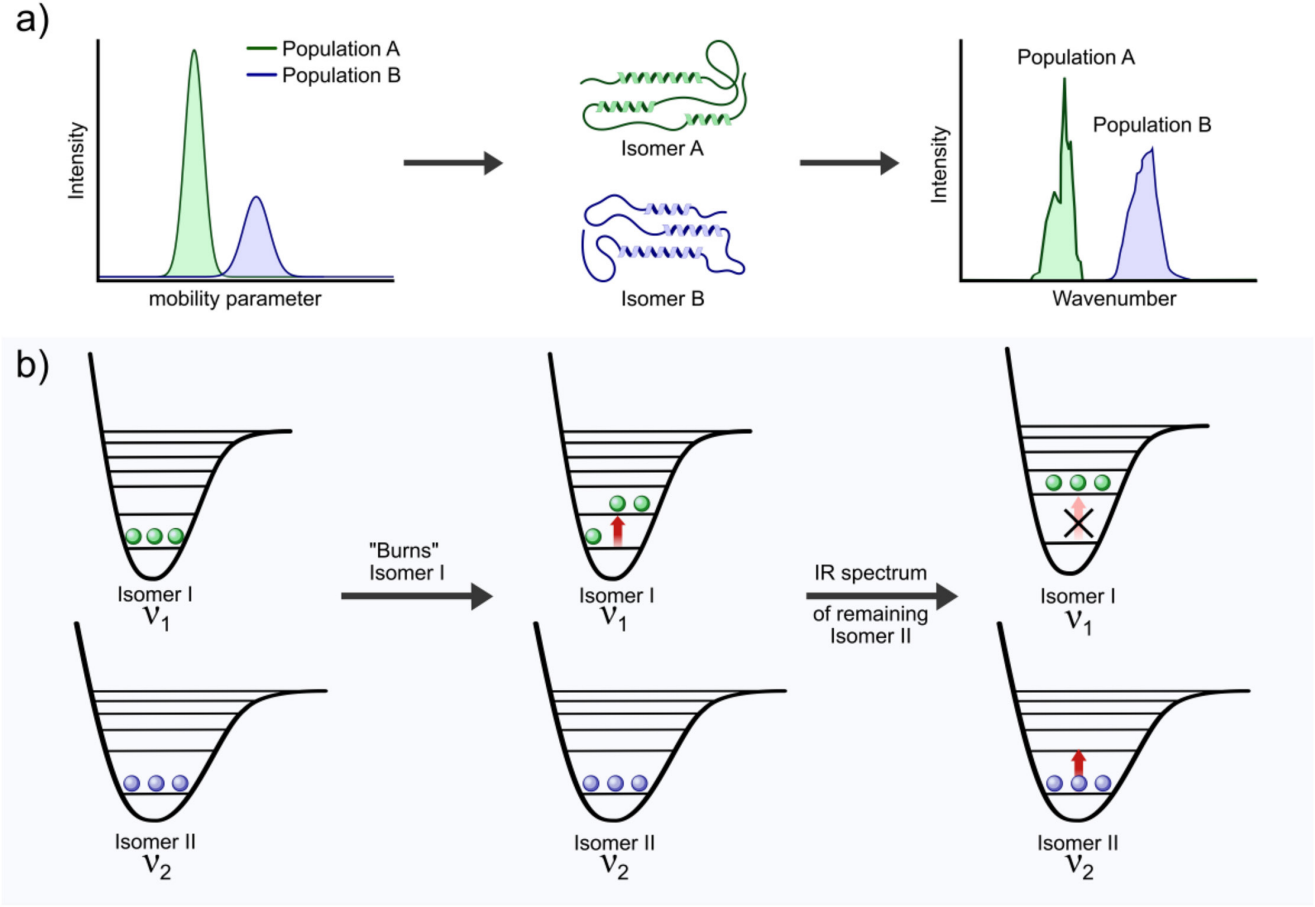
(a) Schematic representation of an ion mobility–ion spectroscopy coupled approach, showing that two isomeric populations can be separated and the population specific spectra can be acquired. (b) Representation of a hole‐burning experiment in the IR range where the ground state population of isomer 1 is depleted by irradiating a photon absorbed by this isomer at a specific *ν*_1_, allowing the remaining population to absorb further photons that will lead to dissociation by scanning *ν*_2_.

IRMPD spectroscopy has also been used as a capable qualitative analytical tool in the identification of isomeric drugs [4, 71, 72]. Kranenburg et al. [71] recorded infrared spectra of drugs of abuse from 600 to 1800 cm^−1^ for the identification of the so‐called novel psychoactive substances (NPS). These substances differ from well‐known drugs of abuse such as MDMA and other amphetamines due to small changes in their structure with unknown health consequences that do not significantly alter the psychoactive effects but might change the legal status of the drugs in different countries.

In that scope, the authors were able to initially verify the applicability of IRMPD spectroscopy for this task by showing how it was possible to successfully record spectra of multiple fluoroamphetamine (FMA), 3,4‐methylenedioxyamphetamine (MDA), and 3,4‐methylenedioxymethamphetamine (MDMA) isomers that revealed distinct vibrational signatures allowing for their easy identification.

In a more applied scenario, the authors then analyzed confiscated street samples of unknown mixtures of drugs. Classic techniques used routinely for this task fell short where IRMPD succeeded. Gas‐chromatography coupled to mass spectrometry (GC‐MS) was unable to achieve full identification of the species as score comparison to the databases did not allow for full differentiation between methyl‐methcathinone (MMC) isomers, while FT‐IR rendered a spectrum with overlapping signals because the samples are mixtures.

With the assistance of DFT computational simulations for harmonic vibrational spectra, the unknown substances could be easily identified without reference standard, showcasing the prospects of action spectroscopy of mass‐selected ions for forensic applications.

Double‐resonance hole‐burning schemes, as those developed in the context of UV‐based cold mass‐selected ions, can also be implemented in a broader sense [73, 74, 75, 76]. The same underlying logic, adapted to an all‐infrared implementation on a single FEL, was applied by van Geenen et al. [77] when they revisited the FMA isomers with two‐color IR–IR double‐resonance spectroscopy (IR^2^MS^3^). This approach was possible due to the 3D ion trap's capability of storing ions for long periods of time and performing multiple MS/MS steps, as represented in Figure 3b.

For each point of the spectral measurement, a fixed wavelength was selected on the FEL for a first step to extinguish a whole population of ions, which had this exclusive vibrational signature. In the following step, all fragments were then ejected from the trap and only the remaining precursor ions (corresponding now to all other populations not removed by the burning step) were kept isolated. The FEL was then tuned to another wavelength to measure that point of the IRMPD spectrum and after completion returned to the wavelength of the burning.

The authors were successful in employing this approach to confirm that in a given mixture of FMA isomers, only two were present. That was possible by simple comparison of obtained IR spectra for the remaining population of ions after burning of the other one with reference spectra obtained for 2‐FMA, 3‐FMA and 4‐FMA, showcasing specific approaches can reveal further information about composition of mixtures, not even demanding computational chemistry assistance.

Although use of helium nanodroplets as spectroscopical matrix dates at least from the 1990s [78], it is only in the 2010s [79, 80] that its coupling with mass selection from molecular ions generated by electrospray started, bringing the technique to the same level of applicability as the other techniques of action spectroscopy mentioned so far [81, 82]. Nowadays, this technique is shown as an interesting and relatively new path for mass‐selected ion spectroscopy.

Walton‐Doyle et al. [83] took on a pair of steroids that differ only in the arrangement of their oxygen functionalities, acquiring high‐resolution infrared spectra of aldosterone and cortisone in the fingerprint region. The bands between 800 and 1800 cm^−1^ were compared with theoretical calculations to identify gas‐phase protonation sites and tautomers. According to the authors, the differentiation of steroid isomers creates the possibility of use for diagnostics and anti‐doping scenarios.

### 3.3 Reactivity and Catalysis

Ion action spectroscopy has also been successfully implemented in the study of reactive and elusive reaction intermediates [3, 84], including organocatalytical reactions [85, 86, 87], metal‐based catalysts [88], polymerization [89], electrochemical‐mediated species [90], and many others. In these cases, ion spectroscopy is particularly advantageous due to one of its major strengths: the ability to isolate transient species directly from the reaction medium without the need for further purification, which could otherwise result in the decomposition or loss of these highly reactive intermediates.

Machida and coworkers [84] caught a species that does not survive isolation, identifying short‐lived oxidation products of phenylhydrazines by UVPD spectroscopy at around 4 K (Figure 4a).

**FIGURE 4:**
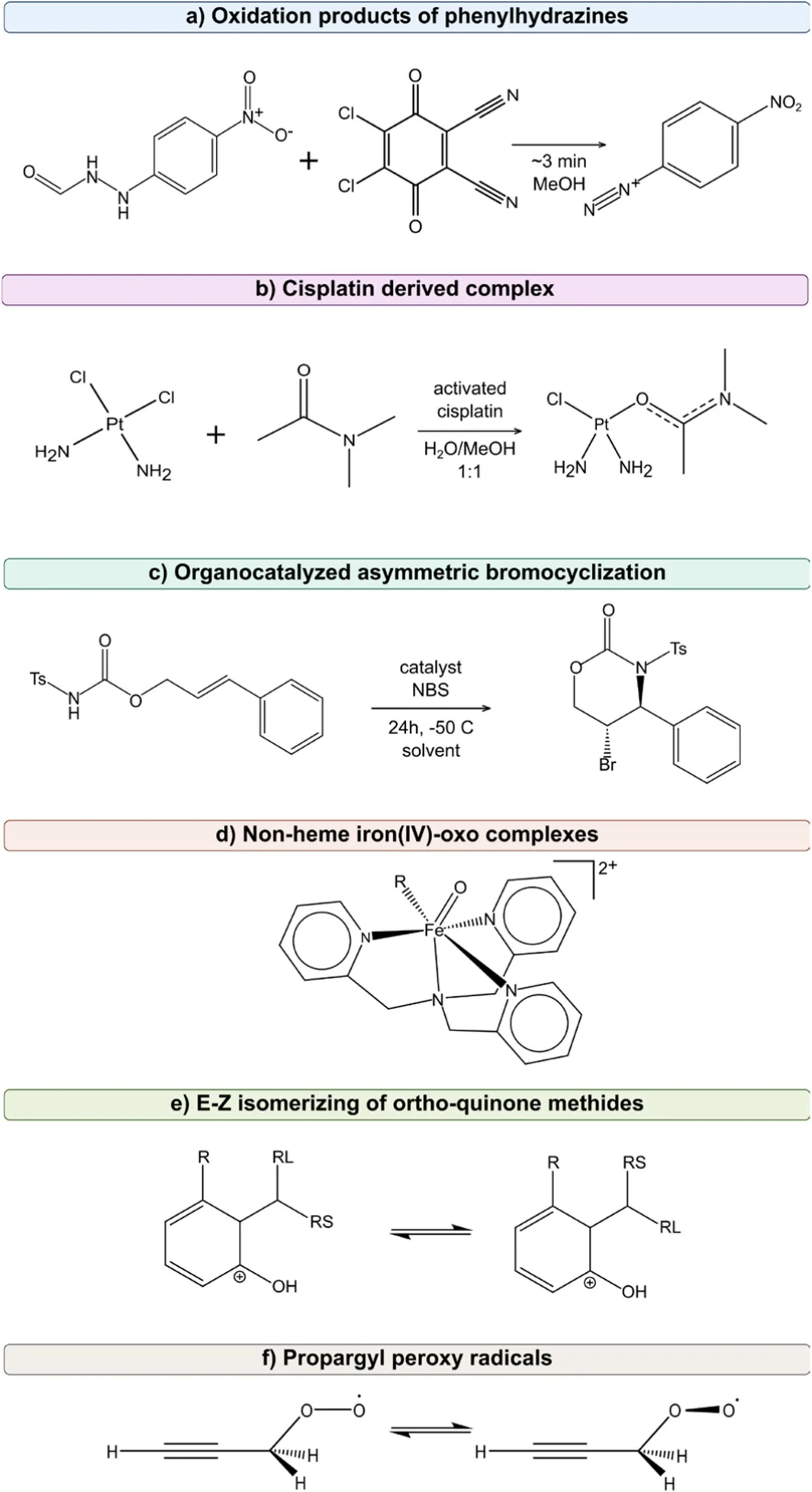
Adapted chemical schemes representative of the systems studied by action spectroscopy discussed in this section. (a) 2‐(4‐nitrophenyl)hydrazinecarboxaldehyde (NHCA), 2,3‐dichloro‐5,6‐dicyano‐p‐benzoquinone (DDQ) and the diazonium cation intermediate observed. (b) Cisplatin and dimethylacetamide (DMA) and the cisplatin‐derived complex. (c) O‐cinnamyl tosyl carbamate and bromoaminocyclization heterocycle product. (d) Derived Iron (IV)‐oxo complex, with R being a labile ligand, such as CH_3_CN, trifluoromethylsulfonate, ArI and ArIO. (e) protonated E/Z‐isomers of ortho quinone methides. (f) Propargyl radical products from the reaction with O_2_ inside He nanodroplets: trans‐acetylenic and gauche‐acetylenic.

The authors were able to identify a cationic intermediate, compatible with one of the species on the plausible reaction route. The UVPD spectra of the intermediate presented two bands, at around 33 900 and 38 600 cm^−1^, consistent with the band positions previously reported in solution for the benzene diazonium salts and nitrobenzene in solution. The experimental data were compared with theoretical calculations conducted using the proposed intermediate structure and the frequencies of the absorption bands could be successfully correlated, corroborating the presence of the diazonium intermediate.

Corinti et al. [91] probed the interaction of cisplatin with small molecules mimicking biologically relevant functional groups (acetamide, dimethylacetamide, urea and thiourea) and their reactivity, combining IRMPD spectroscopy in the fingerprint and XH stretching regions with DFT calculations (Figure 4b). In the four‐coordinate cis‐[PtCl (NH_3_)_2_(L)]^+^ complexes, the missing free C=O stretch predicted for the N‐bound isomers places platination at the carbonyl group. ESI also delivered formally five‐coordinate cis‐[PtCl (NH_3_)_2_(H_2_O)(L)]^+^ ions, whose calculated PES has two minima, the lower one corresponding to the substitution product solvated by water. The absence of the external‐water asymmetric stretch near 3700 cm^−1^ assigns the sampled population to the higher‐energy *cis*‐[PtCl (NH_3_)_2_(H_2_O)]^+^·L adduct, trapped within a kinetic window that thiourea falls outside of, because no five‐coordinate adduct survives to be sampled in that case.

Our group has employed IRMPD spectroscopy to study the reaction mechanism of organocatalyzed asymmetric bromocyclizations (Figure 4c) [86]. The cyclization of *O*‐allyl carbamates mediated by NBS and mediated by *Cinchona*‐based organocatalysts was proposed to take place via different isomeric intermediates, where the activation of the catalyst upon bromide transfer was identified to take place at the thio‐carbonyl sulfur atom. The active catalytic moiety hydrogen bond network that drives the enantioselectivity was revealed by the vibrational spectroscopy of two isomeric intermediates.

Messenger tagging ion spectroscopy has likewise been applied to reactive intermediates. Tripodi and coworkers [92], for example, showed subtle ligand substitutions shifting the reactivity of nonheme iron(IV)‐oxo complexes in ways the calculations did not anticipate, probing them by vibrational tagging photodissociation ion spectroscopy with helium (IRPD, Figure 4d). The authors employed a flow reactor to form the desired complex inline before the MS and spectroscopic analysis. Their results show that the subtle differences in the ligands induce reactivity changes that were not captured by DFT calculations. A similar approach was employed by the same group to allow evaluation of electrochemically generated species by ion spectroscopy [93].

Mayer and Asmis [94] coupled a chip reactor directly to a messenger tagging IRPD‐capable mass spectrometer. The instrument was used to evaluate the Hantzsch cyclization, allowing the differentiation of three distinct isomeric intermediates. They also evaluated the performance of the system for acquiring isomer‐resolved kinetic data and *E‐Z* isomerizing of ortho‐quinone methides (Figure 4e) as a proof‐of‐concept.

Helium nanodroplet action spectroscopy has also found application in the study of radical reactions [95, 96]. Moradi and coworkers [95] entered a long‐running dispute over the entrance channel of the propargyl + O_2_ reaction, embedding the radicals in helium nanodroplets (HENDI‐IR) to follow their reaction with oxygen (Figure 4f) that, after the reaction, were ionized by electron impact. By using difference mass spectra, where the ion signals during laser irradiation and laser off are compared, the authors showed different IR absorption at the 3320–3335 cm^−1^ range for the 1‐butyn‐4‐nitrite precursor, the propargyl radical, and the propargyl peroxy radicals formed after reaction with O_2_, later identified by the full IR spectrum found to be in accordance with simulated vibrational absorptions. Moreover, the authors were able to identify a possible conformational interconversion of the product species by the presence of characteristic CH_2_ bands.

### 3.4 Complex Matrices and Hyphenated Techniques

Most of the examples discussed so far were selected to illustrate the structural questions that action ion spectroscopy can answer, but a distinct and arguably more demanding class of applications concerns samples in which the analyte of interest is a minor component of a complex and largely uncharacterized matrix. In such cases, the analytical difficulty is no longer restricted to distinguishing candidate structures but extends to obtaining a sufficient and clean ion population for the spectroscopic measurement in the first place. A growing body of work demonstrates that this is achievable, allowing the techniques discussed here to be transferred to laboratories outside physical chemistry.

Environmental and agricultural chemistry provide clear illustrations. Infrared ion spectroscopy has been applied to the identification of transformation products [97] to the characterization of contaminants in river water [98], and to the structural elucidation of degradation and biotransformation products of agrochemicals [99, 100], including in the context of the design of more readily degradable active ingredients [101]. As in many of these examples the analytes are frequently unavailable as reference standards, and differ from one another by regiochemistry or by the position of a single functional group, which is precisely the situation in which accurate mass and fragmentation pattern can be least informative.

Clinical and food chemistry applications follow the same logic. The identification of metabolic biomarkers directly in body fluids has been demonstrated for inborn errors of metabolism [102, 103], where the diagnostic value of the measurement depends on distinguishing isomeric metabolites that co‐elute and share a fragmentation pattern. Comparable demands arise in the characterization of human milk oligosaccharides [104], where linkage and anomeric configuration determine biological function. Beyond the life sciences, infrared ion spectroscopy has also been used in materials characterization, for example in the analysis of degradation products in OLED displays [105].

A recurring feature of these studies is that they are usually performed in combination with liquid chromatography. In the model systems discussed earlier in this section, separation is used mainly to isolate isomers prior to interrogation. In real matrices the more pressing requirement is different as chromatographic separation needs to lower matrix effects, limiting ion suppression and isobaric interferences.

Nevertheless, the use of LC‐MS coupled to action spectroscopy is still challenging, due to the incompatible timescale of the techniques, because the action spectroscopy, which generally takes minutes, would have to be carried out during the elution of the species of interest, which occurs during seconds.

This has been approached by Van Outersterp and coworkers [6] by employing HPLC‐MS to obtain separated fractions of the target ions, in this case, hyperlysinemia related metabolites, for later carrying IRMPD at the fingerprint region. By comparing the experimental infrared spectra of the targeted metabolites to quantum‐chemical predicted IR spectra of suitable potential metabolites from the Human Metabolome Database (HMDB) [106, 107, 108, 109], the authors were able to perform the reference‐standard‐free identification of the target metabolite.

Later this methodology was adapted to allow online acquisition of infrared action ion spectroscopy by heartcutting liquid chromatography, combining two sample loops controlled by two multichannel valves, in which the target species can be stored and later injected into the mass spectrometer at rates compatible with IRMPD experiments without the need of previous offline HPLC fraction collection [5].

It should be noted that recently IMS, in its many flavors [110], has been interfaced with LC separation to a greater extent, acting as an orthogonal dimension of separation that enhances the information obtained by MS [111, 112, 113] Compared with LC‐based strategies, IMS allows for faster separation and can be easily coupled to ion spectroscopy techniques [49, 114]. Therefore, LC, IMS, and ion spectroscopy can also be applied together to allow even greater structural characterization of target species [28, 115].

## 4 Commercial Instruments and User Facilities

The practical implementation of gas‐phase ion spectroscopy has been significantly facilitated by advances in both commercial instrumentation and the availability of dedicated user facilities [116, 117].

Action ion spectroscopy experiments have been implemented across a wide range of platforms, with the choice of instrument governed primarily by the required ion confinement time, mass resolution, and compatibility with the irradiation geometry. Ion trap‐based instruments, including quadrupole ion traps (QIT), linear ion traps (LIT), and Fourier‐transform ion cyclotron resonance (FT‐ICR) instruments, are particularly well suited because they allow extended irradiation periods and efficient trapping of both precursor and fragment ions [36, 118, 119, 120]. Orbitrap‐based systems have also been adapted for photodissociation experiments, combining high mass accuracy with the flexibility to incorporate external laser irradiation [121, 122, 123, 124]. Time‐of‐flight platforms have also been employed [57, 63, 66, 125]. The extent to which standard commercial mass spectrometers can be adapted for action ion spectroscopy is one of the most significant practical developments of the past decade, and a driver of the technique's expanding accessibility [118, 119, 126].

Commercial ion trap‐based implementations, either using QIT or linear ion traps, allow minimal modifications to instruments to be made, keeping the robustness of the commercial platform, especially sensitivity and ease of use, and native MS^n^ capabilities positioning these systems as prominent entry‐level platforms for ion action spectroscopy [36, 118].

Furthermore, readily available commercial instruments can now be purchased for easily coupling to radiation sources [63, 127, 128], including ion mobility and cryogenic capabilities [37], further helping in the adoption of ion action spectroscopy by a wider user base. Despite the previous discussion on the imposed limits of tabletop radiation sources for spectroscopy, the past decade saw tabletop Quantum Cascade Laser (QCL) systems being used to perform IR action spectroscopy in the 935–1600 cm^−1^ range at room temperature [129], illustrating that the instrumental barrier is being reduced.

The consolidation of these commercial platforms has recently reached the point at which turnkey operation outside a research laboratory is possible. A complete infrared ion spectroscopy system built from a commercial mass spectrometer and a commercial laser source has been installed and operated within a pharmaceutical company for routine structural elucidation [130], which represents a meaningful milestone. Alongside this, commercial service providers now offer structural identification by action ion spectroscopy [131], so that access to the technique no longer requires either instrument ownership or a facility proposal.

User facilities remain, nevertheless, an important and in some respects unmatched route of access [116, 117]. Beyond the obvious point that the infrastructure already exists and need not be replicated, FEL‐based facilities provide continuous tunability across the full fingerprint region at photon fluences that no benchtop source currently matches, which remains decisive for strongly bound ions and for systems with small absorption cross‐sections.

## 5 Conclusions

Gas‐phase action ion spectroscopy has matured into a versatile analytical approach, capable of addressing structural questions that remain out of reach for conventional mass spectrometry. As illustrated throughout this review, the four representative techniques discussed offer complementary capabilities and allow different classes of structural features to be evaluated.

The application examples presented here were deliberately chosen to reflect the problems for which ion spectroscopy provides analytical value, from biomolecular and disease‐related molecules to pharmaceutical, reactivity and catalytic studies.

The integration of ion spectroscopy with separation techniques such as LC and IMS further expands its practical scope, enabling online structural characterization within analytical workflows that are already familiar to the broader MS community. Simultaneously, the growing availability of benchtop laser sources and commercially adapted MS platforms is progressively removing the instrumental barriers that historically limited access to dedicated physical chemistry laboratories.

## Conflicts of Interest

The authors declare no conflicts of interest.

## Data Availability

Data sharing not applicable to this article as no datasets were generated or analysed during the current study.
